# Acceptability and usefulness of the EORTC ‘Write In three Symptoms/Problems’ (WISP): a brief open-ended instrument for symptom assessment in cancer patients

**DOI:** 10.1186/s12955-024-02244-z

**Published:** 2024-03-26

**Authors:** Leslye Rojas-Concha, Juan Ignacio Arrarrás, Thierry Conroy, Tara Chalk, Monica Guberti, Bernhard Holzner, Olga Husson, Dagmara Kuliś, Omar Shamieh, Claire Piccinin, María José Puga, Gudrun Rohde, Mogens Groenvold, on behalf the EORTC Quality of Life Group

**Affiliations:** 1 Department of Geriatrics and Palliative Medicine GP, Bispebjerg and Frederiksberg Hospitals, Palliative Care Research Unit, University of Copenhagen, Copenhagen, Denmark; 2 Servicio Navarro de Salud, Navarra Institute for Health Research (IdiSNA), Pamplona, Spain; 3 Medical Oncology Department, Institut de Cancérologie de Lorraine, Vandoeuvre-Lès-Nancy, France; 4 Supportive Oncology Research Team, Mount Vernon Cancer Centre, East and North Hertfordshire NHS Trust, Northwood, HA6 2RN UK; 5 Azienda Unità Sanitaria Locale –IRCCS di Reggio Emilia, Reggio Emilia, Italy; 6 University Hospital of Psychiatry II, Medical University of Innsbruck, Innsbruck, Austria; 7 Department of Medical Oncology, Netherlands Cancer Institute – Antoni van Leeuwenhoek, Amsterdam, The Netherlands; 8 Department of Surgical Oncology, Erasmus University Medical Center, Rotterdam, The Netherlands; 9 Quality of Life Department, EORTC, Brussels, Belgium; 10 Department of Hospice, Palliative Medicine, King Hussein Cancer Center, Amman, Jordan; 11 Unidad Alivio del Dolor y Cuidados Paliativos, Hospital Base Valdivia, Valdivia, Chile; 12 Faculty of Health and Sport Sciences, University of Agder, Kristiansand, Norway; 13 Department of Clinical Research, Sorlandet Hospital, Kristiansand, Norway; 14 Department of Public Health, Section of Health Services Research, University of Copenhagen, Copenhagen, Denmark; 15 Université de Lorraine, Inserm INSPIIRE, Nancy, France

**Keywords:** Acceptability, Symptom assessment, Cancer, Palliative care, Prevalence, Quality of Life

## Abstract

**Background:**

The use of open-ended questions supplementing static questionnaires with closed questions may facilitate the recognition of symptoms and toxicities. The open-ended ‘Write In three Symptoms/Problems (WISP)’ instrument permits patients to report additional symptoms/problems not covered by selected EORTC questionnaires. We evaluated the acceptability and usefulness of WISP with cancer patients receiving active and palliative care/treatment in Austria, Chile, France, Jordan, the Netherlands, Norway, Spain and the United Kingdom.

**Methods:**

We conducted a literature search on validated instruments for cancer patients including open-ended questions and analyzing their responses. WISP was translated into eight languages and pilot tested. WISP translations were pre-tested together with EORTC QLQ-C30, QLQ-C15-PAL and relevant modules, followed by patient interviews to evaluate their understanding about WISP. Proportions were used to summarize patient responses obtained from interviews and WISP.

**Results:**

From the seven instruments identified in the literature, only the free text collected from the PRO-CTAE has been analyzed previously. In our study, 161 cancer patients participated in the pre-testing and interviews (50% in active treatment). Qualitative interviews showed high acceptability of WISP. Among the 295 symptoms/problems reported using WISP, skin problems, sore mouth and bleeding were more prevalent in patients in active treatment, whereas numbness/tingling, dry mouth and existential problems were more prevalent in patients in palliative care/treatment.

**Conclusions:**

The EORTC WISP instrument was found to be acceptable and useful for symptom assessment in cancer patients. WISP improves the identification of symptoms/problems not assessed by cancer-generic questionnaires and therefore, we recommend its use alongside the EORTC questionnaires.

**Supplementary Information:**

The online version contains supplementary material available at 10.1186/s12955-024-02244-z.

## Introduction

Cancer patients suffer from many physical and psychosocial problems that require early detection and treatment, regardless of curability of the disease or disease stage [1]. The EORTC Core Quality of Life Questionnaire (QLQ-C30) is one of the most commonly used, validated and translated questionnaires to assess symptoms and quality of life in cancer patients [2–4]. It can be supplemented by disease-specific modules [2]. An abbreviated version of this questionnaire, the EORTC Quality of Life Questionnaire Core 15 Palliative Care (QLQ-C15-PAL), was developed for patients in palliative care [5] and has also been successfully validated in several countries [6–9]. However, static questionnaires with closed questions cannot be expected to cover all symptoms/problems experienced by cancer patients and therefore, the use of open-ended questions supplementing these questionnaires may facilitate the recognition of symptoms and toxicities [10].

A brief supplementary instrument named WISP (Write In three Symptoms/Problems) was developed in Denmark for use alongside the QLQ-C15-PAL. This is an open-ended question allowing patients to report and rate the severity of up to three additional symptoms/problems not included in the QLQ-C15-PAL [11]. The first study published on WISP showed that a third (33%) of the 5,447 patients answering the QLQ-C15-PAL at admittance to Danish specialist palliative care in 2016 reported at least one symptom/problem using WISP, totally 2,796 symptoms/problems added via WISP. Of these, 64% were not covered by the QLQ-C15-PAL, 25% were already covered and 11% were diagnoses or responses that could not be coded [11]. These findings demonstrate that adding WISP to the original QLQ-C15-PAL improves the recognition of symptoms/problems not measured by this questionnaire.

To evaluate the acceptability and usefulness of WISP to cancer patients in general (not just those receiving palliative treatments), we conducted a cross-sectional study involving patients receiving both active and palliative care/treatment from European and non-European countries.

## Methods

This study was conducted in three steps: 1) literature search on validated instruments using open-ended questions in cancer patients, 2) translations of WISP following the EORTC Quality of Life Group (QLG) Translation Procedure [12] and 3) pre-testing the translated WISP alongside the EORTC questionnaires and expanding the qualitative part with structured patient interviews to identify potential comprehension problems, following the EORTC QLG Module Development Guidelines (Phase 3.a) [13]. These steps are described in further detail below.

### Literature search

We performed a literature search on validated instruments with open-ended questions for cancer populations since 1990. The following keywords were searched in PubMed and CINAHL in March–April 2020: (("Patient-reported outcomes" OR "Patient-reported outcome measurements" [MeSH] OR "EORTC-QLQ" OR "systematic assessment") AND (open-ended)) AND ("Terminally ill" [MeSH] OR "advanced cancer" OR "Neoplasms" [MeSH])). When instruments were identified, we contacted the corresponding authors to ask about their experience collecting data with open-ended questions and which coding system they used.

We also contacted all EORTC QLG members asking whether they had knowledge of instruments including open-ended questions used in cancer patients and/or experience performing data analysis for these instruments.

### Study population

For the pre-testing of WISP, we planned to recruit cancer patients from at least 6 countries (at least one English-speaking country and one non-European language country) to assess the WISP instrument in a cross-cultural context [13]. In each included country, we planned to recruit 20 patients receiving diverse cancer treatments; 10 patients should be from an oncology setting (5 patients receiving chemotherapy/radiotherapy and 5 receiving immune/targeted treatment in hospital departments), and 10 patients from a palliative care setting (i.e., receiving palliative care/treatment in a palliative care service, hospice or hospital department).

Inclusion criteria were: 1) having knowledge of the cancer diagnosis, 2) being at least 18 years old, 3) undergoing active antineoplastic treatment or palliative care/treatment, 4) being a native speaker of the country's language, 5) being mentally and physically able to participate, and 6) providing informed consent.

### Translation

**Fig. 1:**
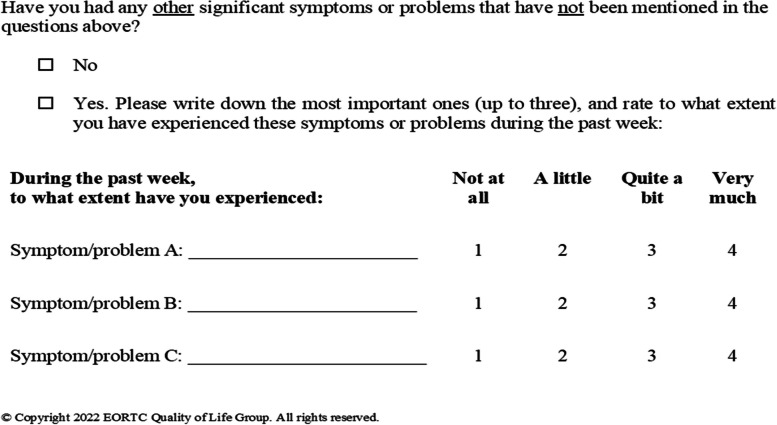
English version of the WISP instrument

The EORTC QLG Translation Unit made forward/backward translations of the original WISP in Danish into English (Fig. 1) and seven additional languages for cultural adaptation. These translated versions of WISP were pilot-tested in each country by asking five cancer patients in active or palliative treatment to review the wording of the instrument and discuss whether the translated version was difficult to answer, confusing or upsetting [12].

### Procedure for the pre-testing of WISP and patient interviews

From November 2022 to June 2023, we pre-tested WISP together with the selected EORTC questionnaires. Patients in active treatment completed the QLQ-C30 + a relevant module according to the patient’s diagnosis if available + WISP, whereas patients in palliative care/treatment completed the QLQ-C15-PAL + WISP.

After patients completed the questionnaires and reported at least 1 symptom/problem using WISP, they were invited to participate in semi-structured interviews to collect information on their understanding and acceptability of WISP, as well as the usefulness of WISP for their reporting of ‘additional symptoms/problems not included in the questionnaire they just completed’ [13]. The interviews were conducted in person by local researchers in the patients' native language, were summarized by the local researcher and not audiotaped, and lasted approximately 15–20 min.

### Questionnaires

The QLQ-C30 comprises 30 items distributed in five functional scales (physical, emotional, role, cognitive and social functioning); three symptom scales (fatigue, pain, nausea/vomiting); two global scales (global health and quality of life) and six single items (insomnia, dyspnea, constipation, lack of appetite, financial difficulties and diarrhea) [4]. The QLQ-C15-PAL consists of half of the items of the QLQ-C30 containing only those items that are most relevant for patients in palliative care [5]. In both EORTC questionnaires (+ module), items are rated on 4-point Likert scales that range from 1 (not at all), 2 (a little), 3 (quite a bit) to 4 (very much), except for global health/quality of life scales rated from 1 (very poor) to 7 (excellent) [4, 5].

WISP consists of a single item asking patients to report up to three symptoms/problems not mentioned in the questionnaire preceding it (i.e., QLQ-C15-PAL or QLQ-C30 + module). Responses on WISP use the same 4-point rating scale as the EORTC questionnaires [11]. This study only reports data collected on WISP.

### Statistical analyses

Patient characteristics were expressed as proportions and compared between patients in active and palliative care/treatment using Chi-square tests (significance level of 0.05).

Qualitative responses from patient interviews were categorized and summarized according to patient treatments. We calculated the proportion of patients adding information about their symptoms/problems and the proportion of patients rating WISP as difficult, annoying, confusing or upsetting to answer.

WISP responses that were rated at least as 2 (a little) were coded using a coding system of 61 symptom/problem categories developed in Denmark to analyze WISP data reported by patients in specialized palliative care [11, 14, 15]. This coding system was developed by grouping the WISP qualitative responses into categories; for example, ‘back pain’ was coded as ‘pain’. If a symptom/problem was not covered by the QLQ-C15-PAL, new codes were established using a list of 48 physical and psychological symptoms developed by Homsi et al., which investigated symptoms reported by palliative patients using open-ended questions versus those systematically assessed [16]. We created extra codes if a symptom/problem did not match any existing category [11]. The prevalence of symptoms/problems reported on WISP was calculated for all cancer patients and for patients receiving different types of treatment (active treatment vs. palliative care/treatment). We calculated the severity as the proportion of symptoms/problems rated as ‘a little’ (mild), ‘quite a bit’ (moderate) and ‘very much’ (severe). Statistical analyses were conducted using the IBM SPSS Statistics 28.

## Results

### Literature search

A total of 35 studies were identified in the literature search (i.e., 25 in PubMed, and10 in CINAHL) and eight studies were suggested by the EORTC QLG members. Based on these results, we identified seven instruments validated in cancer patients that included open-ended questions in their design, but the studies did not include data collected in the open-ended questions.

From June to July 2020, we contacted the corresponding authors of the Edmonton Symptom Assessment System (ESAS) [17], the Memorial Symptom Assessment Scale (MSAS) [18] and its Short Form MSAS-SF [19], the Patient-Reported Outcomes version of the Common Terminology Criteria for Adverse Events (PRO-CTCAE) [20, 21], the EORTC Quality of Life Questionnaire—Lung Cancer Module (QLQ-LC29) [22], the Integrated Palliative care Outcome Scale (IPOS) [23] and the EORTC Quality of Life Questionnaire—Breast Cancer Module (QLQ-BR45) [24].

The authors’ responses showed that a small number of them have collected data from the open-ended questions, but the only analysis and publication was the free text collected from the PRO-CTCAE on the reporting of symptomatic adverse events in three cancer clinical trials [21]. For further details, see Supplementary Table 1.

### Study population

In total 161 cancer patients from 8 countries (Austria, Chile, France, Jordan, the Netherlands, Norway, Spain and the United Kingdom) completed the WISP instrument and were included in this study. Comparisons of background characteristics between patients in active treatment (*n* = 80) and palliative care/treatment (*n* = 81) showed that the distribution of their characteristics was not significantly different, except for diagnosis, type of service and current treatment. The most frequent diagnoses among patients in active treatment were lung and breast cancer and these patients were generally treated with chemotherapy in an oncology department at hospitals, whereas the most frequent diagnosis for patients in palliative care/treatment was cancer in the digestive system, and most received symptom control in palliative care services (Table 1).

**Table 1:** Sociodemographic and clinical characteristics of 161 cancer patients included in the study

Characteristics	Patients in active treatment (*n* = 80)		Patients in palliative care/treatment (*n* = 81)		
	N	%	N	%	*p* value
Sex					0.134
Men	35	43.8	45	55.6	
Women	45	56.3	36	44.4	
Age (years)					0.830
18–39	1	1.3	1	1.2	
40–49	11	13.8	7	8.6	
50–59	14	17.5	14	17.3	
60–69	20	25.0	22	27.2	
70–79	29	36.3	28	34.6	
80 +	5	6.3	9	11.1	
Civil status					0.116
Single	12	15.0	7	8.6	
Married /cohabiting	65	81.3	65	80.2	
Other (widow/divorced/separated)	3	3.8	9	11.1	
Residence					0.305
Private (flat, house, etc.)	77	96.3	80	98.8	
Other (Nursing home, homeless, etc.)	3	3.8	1	1.2	
Education					0.477
Primary education or lower	9	11.3	6	7.4	
Secondary education	28	35.0	35	43.2	
Higher education	43	53.8	40	49.4	
Diagnosis (ICD-10)					0.011
Head and neck (C00-C14, C32)	1	1.3	7	8.6	
Digestive system (C15-17 & C22 + 25)	11	13.8	17	21.0	
Colorectal (C18-C20)	15	18.8	10	12.3	
Lung (C33-C34)	20	25.0	15	18.5	
Breast (C50)	16	20.0	9	11.1	
Prostate (C61)	4	5.0	1	1.2	
Multiple myeloma (C90)	9	11.3	5	6.2	
Leukemia (C91-C95)	1	1.3	5	6.2	
Other cancers (all other C codes)	3	3.8	12	14.8	
Type of service					< 0.001
Oncology department (or outpatient clinic)	70	87.5	22	27.2	
Palliative care service	0	0.0	49	60.5	
Internal medicine department	10	12.5	10	12.3	
Patient status					0.211
Outpatient	62	77.5	69	85.2	
Inpatient	18	22.5	12	14.8	
Current treatment					< 0.001
Palliative care (supportive care, symptom control, etc.)	0	0.0	31	38.3	
Chemotherapy	34	42.5	24	29.6	
Endocrine therapy	4	5.0	2	2.5	
Immunotherapy	12	15.0	16	19.8	
Radiation therapy	10	12.5	0	0.0	
Targeted therapy	6	7.5	2	2.5	
Combination strategies (chemotherapy + radiation, targeted therapy or immunotherapy)	14	17.5	6	7.4	
Country					0.824
Austria	10	12.5	10	12.3	
Chile	10	12.5	10	12.3	
France	10	12.5	10	12.3	
Jordan	9	11.3	12	14.8	
Netherlands	9	11.3	10	12.3	
Norway	8	10.0	12	14.8	
Spain	10	12.5	10	12.3	
United Kingdom	14	17.5	7	8.6	

*ICD-10* International Statistical Classification of Diseases and Related Health Problems 10th

### Patient interviews

Table 2 summarize the qualitative answers obtained from patient interviews. Overall, WISP was widely accepted as less than 2% of the patients had difficulties answering WISP or found it annoying or upsetting. Only a few palliative care patients (*n* = 5) commented that they were confused about what type of symptoms they should report using WISP (i.e., physical, psychological, etc.). Among the 27 patients who provided additional comments on WISP (question 7), 8 expressed positive opinions on the usefulness and relevance of WISP for reporting their symptoms/problems not covered by the EORTC questionnaires. Most patients believed that the symptoms/problems they reported on WISP were a consequence of their cancer treatment or the disease itself (75.8%).

**Table 2:** Summary of patient interviews

Questions	Number of patients answering each question (*n* = 161)^a^	Patients in active treatment (*n* = 80)		Patients in palliative care/treatment (*n* = 81)	
		N (%)	Example comments	N (%)	Example comments
1. I can see that you experienced one or more additional symptoms/problems during the past week. Is this correct? Can you tell me about it/them?	Described additional symptoms or problems *n* = 110 (68.3%)	48 (60.0%)	“Burning inside the mouth is causing more or less significant discomfort to eat or drink” (P103) “My incontinence is embarrassing and forcing me to avoid normal activities” (P115) “Because of my sore mouth, I lost the taste” (P136)	62 (76.5%)	“I experienced dizziness in the last 2 months and can’t go out of the house” (P7) “I have trouble controlling urine. At first it limited me a lot. Then they recommended incontinence pads” (P9) “I can no longer walk long distances or carry things” (P53)
2. Do you think that this/these problem/s is/are related to your disease or treatment?	Answered yes, *n* = 122 (75.8%)	61 (76.3%)	“I think it is due to Avastin during the 3 days treatment” (P102) “My weight loss is due to illness and treatment” (P142) “Both additional symptoms are associated with chemotherapy” (P155)	61 (75.3%)	“Tingling only during chemo then it lasts a few days then disappears” (P63) “I lost the taste because of the therapy” (P28) “I heard from another doctor that there is a medicine which helps for being nauseous as a side effect of immunotherapy, wish I heard this earlier” (P51)
3. Did you have difficulty in replying to this question?	Answered yes, *n* = 3 (1.9%)	2 (2.5%)	“I have difficulties talking about my health problems” (P139) “I don’t like questionnaires like this” (P149)	1 (1.2%)	“I don’t know what to do with WISP” (P52)
4. Do you think this question is annoying?	Answered yes, *n* = 1 (0.6%)	1 (1.3%)	“Questionnaires are unnecessary” (P128)	0 (0%)	
5. Do you find this question is confusing?	Answered yes, *n* = 5 (3.1%)	0 (0%)		5 (6.2%)	“I much prefer closed questions” (P1) “The item is not clear about the type of symptoms it is asking about (physical, psychological, emotional, or other symptoms)” (P3) “Unclear what type of symptoms/problems the question means, physical, social, emotional etc.?” (P47)
6. Do you find this question upsetting?	Answered yes, *n* = 2 (1.2%)	1 (1.3%)	“It's upsetting to talk about pain caused by therapy” (P137)	1 (1.2%)	“A little bit, going over and thinking about symptoms/problems is upsetting” (P57)
7. Do you have other comments about this question (e.g., on its usefulness or relevance for reporting symptoms, etc.)?	Answered yes, *n* = 27 (16.8%)	12 (15.0%)	“I found it more difficult to formulate additional symptoms than responding to the ones already written” (P107) “There is too little attention on the theme sexuality, should be incorporated in the questionnaire as this is a big part of someone's quality of life” (P116) “I like to answer questionnaires and be aware of symptoms” (P126) “I liked this question to talk about my symptom load” (P169)	15 (18.5%)	“It’s better to add an explanatory example (e.g., physical, psychological, emotional, etc.)” (P4) “In the questionnaires you have the option yes/no, but sometimes is nice to have space to include "something else" (P16) “I am thankful for this interview and talk about my symptoms” (P52) “Why was numbness not covered by the EORTC questionnaire?” (P54)
8. How would you paraphrase this question? How would you ask it?	Patients who paraphrased WISP, *n* = 36 (22.4%)	15 (18.8%)	“Have you had other symptoms or problems?” (P82) “Please list any symptoms other than those listed above” (P112) “Is there something else you would like to report?” (P120)	21 (25.9%)	“Have you had any other symptoms (physical, psychological, emotional, etc.) that were not mentioned above? (P3) “Have you other distressing symptoms?” (P43) “Have you had other problems/symptoms in your daily life?” (P48)

^a^In questions 1 to 7 patients had the possibility of answering yes or no to each question, and when the answer was yes, they were asked to add comments to their answer

### Prevalence and severity of symptoms and problems reported on WISP

**Fig. 2:**
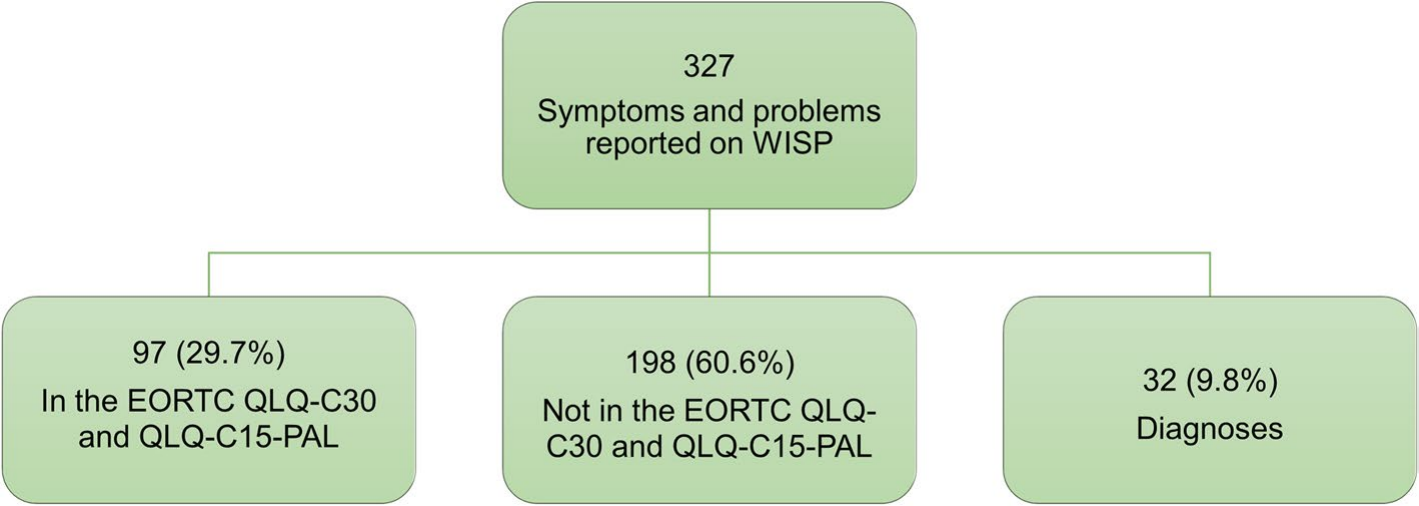
Classification of symptoms and problems reported on the WISP instrument by 161 cancer patients

In total 327 symptoms/problems were reported using the WISP instrument by the 161 cancer patients. Of these, 60.6% were symptoms/problems not covered by the selected EORTC questionnaires, 29.7% were symptoms/problems already covered by the questionnaires and 9.8% were responses coded as diagnoses (Fig. 2). The most frequent diagnoses listed on WISP were mucus (25.0%), infection (9.4%) and respiratory diseases (9.4%) (Supplementary Table 2).

The prevalence of the 295 symptoms/problems covered or not by the selected EORTC questionnaires were grouped into 49 symptom/problem categories and presented for all cancer patients and for patients receiving different types of treatment (Table 3). The most prevalent symptoms/problems not covered by the EORTC questionnaires listed on WISP by all cancer patients were skin problems (16.1%), numbness/tingling (13.7%), dry mouth (9.3%), existential problems (6.2%) and bleeding (5.0%). Skin problems, sore mouth and bleeding were commonly reported by patients in active treatment, whereas dry mouth, numbness/tingling and existential problems were often reported by patients in palliative care/treatment. Pain (19.3%) and impaired emotional function (9.9%) were among the most prevalent symptoms/problems already covered by the EORTC questionnaires. Overall, 78.0% of symptoms/problems were reported as moderate to severe on WISP, where social and speaking problems were among the most severe (Table 4).

**Table 3:** Prevalence of 295 symptoms and problems (grouped into 49 categories) reported on the WISP instrument by 161 cancer patients. Symptoms and problems already covered by the QLQ-C30 and QLQ-C15-PAL questionnaires are in italic

49 symptom/problem categories	All patients (*n* = 161)		Patients in active treatment (*n* = 80)		Patients in palliative care/treatment (*n* = 81)	
	N	%	N	%	N	%
*Pain*	31	19.3	18	22.5	13	16.0
Skin problems	26	16.1	14	17.5	12	14.8
Numbness/tingling	22	13.7	10	12.5	12	14.8
*Impaired emotional function*^b^	16	9.9	9	11.3	7	8.6
*Fatigue*	15	9.3	5	6.3	10	12.3
Dry mouth	15	9.3	1	1.3	14	17.3
*Impaired physical function*^c^	12	7.5	5	6.3	7	8.6
Existential problems	10	6.2	3	3.8	7	8.6
Bleeding	8	5.0	5	6.3	3	3.7
Edema	7	4.3	3	3.8	4	4.9
Dizziness	7	4.3	1	1.3	6	7.4
Itching	7	4.3	4	5.0	3	3.7
Myoclonus^d^	7	4.3	1	1.3	6	7.4
Sore mouth	6	3.7	6	7.5	0	0.0
Other eye symptoms	6	3.7	2	2.5	4	4.9
Heartburn	6	3.7	1	1.3	5	6.2
Dysphagia	5	3.1	2	2.5	3	3.7
Sweats	5	3.1	2	2.5	3	3.7
Weight loss	5	3.1	2	2.5	3	3.7
Shakiness	5	3.1	4	5.0	1	1.2
Incontinence^e^	5	3.1	3	3.8	2	2.5
*Diarrhea*^a^	5	3.1	0	0.0	5	6.2
Headache	4	2.5	1	1.3	3	3.7
Indigestion	4	2.5	2	2.5	2	2.5
*Social problems*^a^	4	2.5	1	1.3	3	3.7
*Economic problems*^a^	4	2.5	2	2.5	2	2.5
Confusion	3	1.9	1	1.3	2	2.5
*Sleeping difficulties*	3	1.9	1	1.3	2	2.5
Cough	3	1.9	1	1.3	2	2.5
Speaking problems	3	1.9	1	1.3	2	2.5
Vision problems	3	1.9	1	1.3	2	2.5
Chills	3	1.9	3	3.8	0	0.0
Sexual problems	3	1.9	1	1.3	2	2.5
Low satisfaction with care	3	1.9	2	2.5	1	1.2
Bloating	3	1.9	1	1.3	2	2.5
Taste change	3	1.9	1	1.3	2	2.5
*Nausea*	2	1.2	1	1.3	1	1.2
Fever	2	1.2	1	1.3	1	1.2
Burning sensation	2	1.2	2	2.5	0	0.0
Urinary problems	2	1.2	1	1.3	1	1.2
Distress in the body	2	1.2	1	1.3	1	1.2
*Dyspnea*	1	0.6	0	0.0	1	1.2
*Lack of appetite*	1	0.6	1	1.3	0	0.0
*Vomiting*^a^	1	0.6	0	0.0	1	1.2
Thirst	1	0.6	0	0.0	1	1.2
Hallucinations^f^	1	0.6	0	0.0	1	1.2
Heaviness	1	0.6	1	1.3	0	0.0
*Concentration problems*^a^	1	0.6	0	0.0	1	1.2
*Reduced memory*^a^	1	0.6	1	1.3	0	0.0
Total	295	100	129	43.9	166	56.5

^a^Symptoms/problems covered by the QLQ-C30 questionnaire only

^b^Including feeling anxious, concerned, irritated and sad

^c^Including balance/coordination problems, muscular weakness, reduced mobility and walking problems

^d^Including muscle cramps and spasms

^e^Including urinary, stool and unspecified incontinence

^f^Including visual, auditory and unspecified hallucinations

**Table 4:** Frequency and severity of 295 symptoms and problems (grouped into 49 categories) reported on the WISP instrument by 161 cancer patients. Symptoms and problems already covered by the QLQ-C30 and QLQ-C15-PAL questionnaires are in italic

49 symptom/problem categories	Symptoms and problems reported on WISP = 295							
	Frequency		Severity					
			Mild		Moderate		Severe	
	N	%	N	%	N	%	N	%
*Pain*	31	10.6	5	16.1	13	41.9	13	41.9
Skin problems	26	8.9	7	26.9	12	46.2	7	26.9
Numbness/tingling	22	7.5	8	36.4	8	36.4	5	27.3
*Impaired emotional function*^b^	16	5.5	2	12.5	5	31.3	9	56.3
*Fatigue*	15	5.1	4	26.7	6	40.0	5	33.3
Dry mouth	15	5.1	5	33.3	7	46.7	3	20.0
*Impaired physical function*^c^	12	4.1	0	0.0	5	41.7	7	58.3
Existential problems	10	3.4	1	10.0	2	20.0	7	70.0
Bleeding	8	2.7	2	25.0	1	12.5	5	62.5
Edema	7	2.4	2	28.6	1	14.3	4	57.1
Dizziness	7	2.4	4	57.1	0	0.0	3	42.9
Itching	7	2.4	2	28.6	1	14.3	4	57.1
Myoclonus^d^	7	2.4	3	42.9	2	28.6	2	28.6
Sore mouth	6	2.0	1	16.7	0	0.0	5	83.3
Other eye symptoms	6	2.0	1	16.7	3	50.0	2	33.3
Heartburn	6	2.0	0	0.0	2	33.3	4	66.7
Dysphagia	5	1.7	0	0.0	2	40.0	3	60.0
Sweats	5	1.7	0	0.0	1	20.0	4	80.0
Weight loss	5	1.7	0	0.0	1	20.0	4	80.0
Shakiness	5	1,7	2	40.0	2	40.0	1	20.0
Incontinence^e^	5	1.7	1	20.0	1	20.0	3	60.0
*Diarrhea*^a^	5	1.7	2	40.0	2	40.0	1	20.0
Headache	4	1.4	0	0.0	1	25.0	3	75.0
Indigestion	4	1.4	0	0.0	1	25.0	3	75.0
*Social problems*^a^	4	1.4	0	0.0	0	0.0	4	100
*Economic problems*^a^	4	1.4	0	0.0	3	75.0	1	25.0
Confusion	3	1.0	2	66.7	1	33.3	0	0.0
*Sleeping difficulties*	3	1.0	1	33.3	1	33.3	1	33.3
Cough	3	1.0	1	33.3	2	66.7	0	0.0
Speaking problems	3	1.0	0	0.0	0	0.0	3	100
Vision problems	3	1.0	1	33.3	2	66.7	0	0.0
Chills	3	1,0	1	33.3	1	33.3	1	33.3
Sexual problems	3	1.0	2	66.7	0	0.0	1	33.3
Low satisfaction with care	3	1.0	0	0.0	2	66.7	1	33.3
Bloating	3	1.0	1	33.3	1	33.3	1	33.3
Taste change	3	1.0	1	33.3	1	33.3	1	33.3
*Nausea*	2	0.7	0	0.0	1	50.0	1	50.0
Fever	2	0.7	1	50.0	0	0.0	1	50.0
Burning sensation	2	0.7	0	0.0	1	50.0	1	50.0
Urinary problems	2	0.7	1	50.0	0	0.0	1	50.0
Distress in the body	2	0.7	0	0.0	0	0.0	2	100
*Dyspnea*	1	0.3	0	0.0	1	100	0	0.0
*Lack of appetite*	1	0.3	0	0.0	1	100	0	0.0
*Vomiting*^a^	1	0.3	0	0.0	0	0.0	1	100
Thirst	1	0.3	0	0.0	1	100	0	0.0
Hallucinations^f^	1	0.3	0	0.0	1	100	0	0.0
Heaviness	1	0.3	0	0.0	1	100	0	0.0
*Concentration problems*^a^	1	0.3	1	100	0	0.0	0	0.0
*Reduced memory*^a^	1	0.3	0	0.0	0	0.0	1	100
Total	295	100	65	22.0	99	33.6	131	44.4

^a^Symptoms/problems covered by the QLQ-C30 questionnaire only

^b^Including feeling anxious, concerned, irritated and sad

^c^Including balance/coordination problems, muscular weakness, reduced mobility and walking problems

^d^Including muscle cramps and spasms

^e^Including urinary, stool and unspecified incontinence

^f^Including visual, auditory and unspecified hallucinations

## Discussion

In this study, we evaluated the open-ended WISP instrument with 161 cancer patients (in active and palliative care/treatment) across eight countries. Our main findings were that WISP showed high acceptability during patient interviews, as a low proportion of patients (2%) found that WISP was difficult to answer. Additionally, WISP proved to be useful in identifying many symptoms/problems (*n* = 198) not covered by the selected EORTC questionnaires.

A total of 327 symptoms/problems were reported using WISP, of which 60.6% were symptoms/problems not covered by the EORTC questionnaires. Among the most prevalent symptoms/problems listed on WISP, skin problems, numbness/tingling, dry mouth, existential problems and bleeding have also previously been reported as frequent symptoms voluntarily reported by advanced cancer patients [11, 16, 25].

We found that the 80 patients in active treatment frequently reported skin problems (17.5%), sore mouth (7.5%) and bleeding (6.3%). The prevalence of skin problems we observed was higher than reported via open-ended questions in other studies (7–14%) by 50 and 69 cancer patients, respectively [26, 27]. This may reflect the fact that not all patients included in those studies were receiving antineoplastic treatment like our patients. Furthermore, the high prevalence of sore mouth and bleeding is in line with the literature showing that these adverse effects are usually reported by patients while receiving chemotherapy and radiotherapy [28, 29].

Patients in palliative care/treatment often reported dry mouth (17.3%), numbness/tingling (14.8%) and existential problems (8.6%). The prevalence of dry mouth and numbness/tingling found in our study was very high compared to the prevalence of dry mouth (1.3%) and numbness/tingling (1.0%) reported earlier by 1,788 palliative care patients using WISP [11] and by 200 palliative patients using an open-ended question before a list of 48 symptoms (1.5% dry mouth; 2.0% numbness/tingling) [16]. Remarkably, our palliative care patients reported existential problems much more frequently than in the previous Danish study using WISP (0.9%) [11]. This difference may reflect that 60% of our palliative patients were also receiving chemotherapy or another combined therapy, and they may have been considering side effects or had more concerns about the future, while palliative patients in the previous study were mainly receiving end of life care [11].

Regarding the symptoms/problems already covered by the EORTC questionnaires, pain (19.3%) and impaired emotional function (9.3%) were among the most prevalent symptoms/problems. This is consistent with previous studies showing that pain is the most common symptom elaborated by cancer patients using open-ended questions, especially when they need to report the location of the pain [16, 25, 27, 30]. The prevalence of impaired emotional function in our study was higher than in the previous studies using WISP (2–3%) [11, 31].

A strength of this study is that we included a diverse sample of cancer patients (i.e., receiving different type of treatments, at different disease stage and from several countries). To our knowledge, WISP is the only open-ended instrument for which experience with coding and analyses of additional symptoms/problems experienced by diverse cancer populations has been reported [11, 31]. Most of the questionnaires with open-ended questions identified in the literature did not have a coding system in place [19, 24] or the answers were barely analyzed [17, 22]. We also confirmed that the previously developed coding system for WISP was efficient, as only two new codes were needed for this study (i.e., sexual problems and low satisfaction with care). However, we know that collecting data using open-ended questions and the work of manually coding responses may be impractical, but this is the first step to provide a brief instrument that can supplement any other EORTC questionnaire to detect those additional symptoms/problems that are important to patients and need to be addressed during the clinician-patient encounter. WISP also has the potential advantage of reducing the burden of patients as compared to completing lengthy questionnaires.

The next steps for the EORTC WISP instrument will be 1) evaluating its usability in clinical trials by collecting relevant symptoms and toxicities experienced by cancer patients, especially in early phase trials when less is known about the potential effects of a cancer treatment and selecting questionnaires/items can be challenging; 2) linking the 63 WISP categories to the 1,060 items in the EORTC Item library to identify missing items and strengthen the WISP coding system based on previous experiences [21, 32], 3) developing a digital solution for WISP with the option of a dropdown list and/or free text, and 4) exploring whether the most frequently reported symptoms/problems on WISP could contribute to the prediction of health outcomes and should be included to static questionnaires.

## Conclusions

The EORTC WISP instrument was found to be acceptable and useful for symptom assessment in cancer patients. As anticipated, distinct differences were seen in the reporting of symptom/problems using WISP between patients in active and palliative care/treatment.

This study confirms the utility of WISP to improve the identification of symptoms/problems not assessed by cancer-generic questionnaires. We therefore recommend its use alongside the EORTC questionnaires to achieve a more comprehensive symptom assessment.

### Supplementary Information

**Supplementary Material 1.**

## Data Availability

The datasets used and/or analyzed during the current study are available from the corresponding author on reasonable request.

## Abbreviations

EORTC: European Organisation for Research and Treatment of Cancer
ESAS: Edmonton Symptom Assessment System
Fig: Figure
IPOS: Integrated Palliative care Outcome Scale
MeSH: Medical Subject Headings
MSAS: Memorial Symptom Assessment Scale
MSAS-SF: Memorial Symptom Assessment Scale-Short Form
PRO-CTCAE: Patient-Reported Outcomes version of the Common Terminology Criteria for Adverse Events
QLG: Quality of Life Group
QLQ-C30: EORTC Core Quality of Life Questionnaire
QLQ-C15-PAL: EORTC Quality of Life Questionnaire Core 15 Palliative Care
QLQ-BR45: EORTC Quality of Life Questionnaire - Breast Cancer Module
QLQ-LC29: EORTC Quality of Life Questionnaire - Lung Cancer Module
QOL: Quality of life
WISP: The Write In three Symptoms/Problems instrument
