# Supplementary material for: Acceptability and usefulness of the EORTC ‘Write In three Symptoms/Problems’ (WISP): a brief open-ended instrument for symptom assessment in cancer patients
Source: Health Qual Life Outcomes. 2024 Mar 26;22:28. doi: 10.1186/s12955-024-02244-z (PMC10964595; doi:10.1186/s12955-024-02244-z)
Supplement: Supplementary file 1 — Supplementary Material 1. [file 12955_2024_2244_MOESM1_ESM.docx]

Supplementary Table 1. Summary of the open-ended questions included in validated instruments for cancer population and comments from their authors about their experience collecting and analyzing data from these questions.

| **Instrument** | **Authors/**  **Year** | **Open-ended question design** | **Number of symptoms**  **Collected** | **Severity ratings** | **Author comments** |
| --- | --- | --- | --- | --- | --- |
| ESAS | Bruera, 1991 | ‘Other problem (for example constipation)’ | Up to one | 0 (no symptom) to 10 (worst possible) | “At the beginning of ESAS use, we had an open assessment of any other symptoms. We decided to stop using it as reported symptoms were rarely action items” |
| MSAS | Portenoy, 1994 | ‘If you had any other symptoms during the past week, please list them below, and indicate how much the symptom had distressed you’ | Up to four | 1 (not at all) to 5 (very much) | The corresponding author did not respond to emails, but Dr Chang, the author of the short form of the MSAS, confirmed that data on the open-ended question from MSAS or MSAS-SF have not been published |
| MSAS-SF | Chang, 2000 | ‘If you had any other symptoms during the past week, please list them below, and indicate how much the symptom had distressed you’ | Up to two | 1 (not at all) to 5 (very much) | “No one has written anything for the open-ended question” |
| PRO-CTCAE | Basch, 2014 | Item ‘other symptoms’: ‘Do you have any other symptoms that you wish to report? Yes/no’. Please list any other symptoms | Up to five | None, mild, moderate, severe, very severe | “Yes, we do collect free text in the PRO-CTCAE software. In a prior study, patients structured their responses with a drop-down function and allowed for free text to be entered. Based on this approach and collected data, we published the following analysis: <https://pubmed.ncbi.nlm.nih.gov/30840079/>” |
| QLQ-LC29 | Koller, 2017 | ‘Were there any symptoms or problems that were not covered by the questionnaire, but relevant for you in the past week?’ | Up to three | 1 (not at all) to  4 (very much) | “A total of 139 symptoms were listed in the open-ended questions by 523 patients at the first assessment, but these data were not published” |
| IPOS | Murtagh & Higginson, 2019 | ‘Please list any other symptoms not mentioned above, and tick one box to show how they have affected you over the past 3 days’ | Up to three | 1 (not at all) to 4 (overwhelmingly) | Authors did not respond to emails |
| QLQ-BR45 | Bjelic-Radisic, 2020 | ‘Were there any symptoms or problems that were not covered by the questionnaire, but relevant for you in the past week?’ | Up to three | 1 (not at all) to  4 (very much) | “We did not collect (extract) data” |

Abbreviations: ESAS=Edmonton Symptom Assessment System; MSAS=Memorial Symptom Assessment Scale; MSAS-SF=Memorial Symptom Assessment Scale-Short Form; PRO-CTCAE=Patient-Reported Outcomes version of the Common Terminology Criteria for Adverse Events; QLQ-LC29= EORTC Quality of Life Questionnaire - Lung Cancer Module; IPOS=the Integrated Palliative care Outcome Scale; QLQ-BR45=EORTC Quality of Life Questionnaire - Breast Cancer Module

Supplementary Table 2. Frequency of 32 responses listed on the WISP instrument and coded as diagnoses.

| **Diagnoses** | **N** | **%** |
| --- | --- | --- |
| Mucus | 8 | 25.0 |
| Infection | 3 | 9.4 |
| Respiratory diseases^a^ | 3 | 9.4 |
| Poor blood circulation | 2 | 6.3 |
| Stiffness | 2 | 6.3 |
| Hypertension | 2 | 6.3 |
| Wound | 2 | 6.3 |
| Teeth and gums problems | 2 | 6.3 |
| Heart problems (not specified) | 1 | 3.1 |
| Diabetes | 1 | 3.1 |
| Fracture | 1 | 3.1 |
| Fungus | 1 | 3.1 |
| Cerebrovascular accident (CVA) | 1 | 3.1 |
| Anemia | 1 | 3.1 |
| Thyroid problems | 1 | 3.1 |
| Menopause | 1 | 3.1 |
| Total | 32 | 100 |

^a^ Including pneumonia and bronchitis
